# Assessing anatomical variations of the inferior mesenteric artery via three-dimensional CT angiography and laparoscopic colorectal surgery: a retrospective observational study

**DOI:** 10.1038/s41598-024-57661-3

**Published:** 2024-03-24

**Authors:** Yuanyi Ding, Botian Zhao, Wenbo Niu, Xuhua Hu, Chenhui Li, Zining Liu, Bin Yu

**Affiliations:** The Second Department of Surgery, The Fourth Hospital of Hebei Medical University, No. 12, Jiankang Road, Shijiazhuang, Hebei People’s Republic of China

**Keywords:** Inferior mesenteric artery, Left colic artery, Laparoscopic surgery, Multiple spiral three-dimensional computed tomography angiography, Colorectal cancer, Colonoscopy, Surgical oncology

## Abstract

To assess the anatomy of the inferior mesenteric artery (IMA) and its branches by reviewing laparoscopic left-sided colorectal cancer surgery videos and comparing them with preoperative three-dimensional computed tomography (3D-CT) angiography, to verify the accuracy of 3D-CT vascular reconstruction techniques. High-definition surgical videos and preoperative imaging data of 200 patients who underwent laparoscopic left-sided colorectal cancer surgery were analysed, and the alignment of the IMA and its branches in relation to the inferior mesenteric vein (IMV) was observed and summarized. The above two methods were used to measure the length of the IMA and its branches. Of 200 patients, 47.0% had the sigmoid arteries (SAs) arise from the common trunk with the superior rectal artery (SRA), and 30.5% had the SAs arise from the common trunk with the left colic artery (LCA). In 3.5% of patients, the SAs arising from both the LCA and SRA. The LCA, SA, and SRA emanated from the same point in 13.5% of patients, and the LCA was absent in 5.5% of patients. The range of D cm (IMA length measured by intraoperative silk thread) and d cm (IMA length measured by 3D-CT vascular reconstruction) in all cases was 1.84–6.62 cm and 1.85–6.52 cm, respectively, and there was a significant difference between them. (p < 0.001). The lengths between the intersection of the LCA and IMV measured intraoperatively were 0.64–4.29 cm, 0.87–4.35 cm, 1.32–4.28 cm and 1.65–3.69 cm in types 1A, 1B, 1C, and 2, respectively, and there was no significant difference between the groups (p = 0.994). There was only a significant difference in the length of the IMA between the 3D-CT vascular reconstruction and intraoperative observation data, which can provide guidance to surgeons in preoperative preparation.

## Introduction

In recent years, the incidence of colorectal cancer has been increasing^1^. With the development of laparoscopic techniques for gastrointestinal oncological surgery, laparoscopic radical resection of colorectal cancer has been increasingly used in clinical practice. Ligation of the inferior mesenteric artery (IMA) is one of the important steps in laparoscopic radical resection of left-sided colorectal cancer, but the ligation location differs based on the requirements of radical operation, the surgeon's proficiency in the surgery, and the understanding of the anatomical relationship between blood vessels and nerves. Nowadays left colon and rectal cancer treatment has been well standardized in laparoscopy, nevertheless, the level of the ligation of the IMA, at the origin from the aorta (high tie) or below the origin of the left colic artery (LCA) (low tie), is still debated^2^. Different ligation sites may have an impact on overall surgical quality and patient prognosis. Although it is debated whether high ligation of the IMA will affect the probability of anastomotic fistula formation^2,3^, it is clear that high ligation will reduce the blood flow of the anastomosis^4–6^. Due to the colonic blood supply being relatively poor at the splenic flexure of the colon and the rectosigmoid junction^7^, some surgeons have performed D3 lymph node dissection and preserved the LCA to maintain the blood supply to the proximal sigmoid colon^8,9^. Compared with high ligation of the IMA, this method requires more surgical techniques and may increase the operative time^10,11^.

Because the two-dimensional laparoscopic views differ considerably from the open view, and lack of tactile sense, the bifurcations and variants of blood vessels during ligation of the mesenteric artery may be misidentified and injured, and blood vessels are more likely to bleed, thereby causing blurred visibility of local tissue and further increasing the difficulty of the operation. At present, the development of new techniques can help surgery, previous studies have confirmed that indocyanine green near-infrared fluoroangiography (NIRF/ICG) can potentially reduce the probability of anastomotic leakage by assessing the degree of intraoperative anastomotic perfusion^12^; three-dimensional laparoscopic surgery can bring a more stereoscopic vision, without increasing the cognitive load^13^. These technologies are conducive to intraoperative vascular anatomy and reduce surgical complications. However, the alignment of the IMA and its branches, the relationship between the IMA and the abdominal aorta (AA), and the association of the LCA with the inferior mesenteric vein (IMV) vary from person to person. It is still very important to predict the anatomical variation of IMA before operation.

Currently, research on the anatomical structure and variation of the IMA has been carried out by three-dimensional computed tomography (3D-CT) angiography. Direct intraoperative measurement is more intuitive and accurate than the 3D revascularization technique. However, no direct comparison of these two methods of study has been made, and the accuracy and importance of vascular 3D reconstruction techniques for surgery need to be urgently addressed. The aims of our study are to analyse the anatomy of the IMA and its branches by reviewing video recordings of laparoscopic left-sided colorectal cancer surgery and using the 3D-CT reconstruction technique and to explore the difference between the two methods, to validate the accuracy of 3D-CT reconstructed vessels.

## Methods

### Patient selection

All patients in this study were from the Second Department of Surgery, The Fourth Hospital of Hebei Medical University (Shijiazhuang, Hebei, People's Republic of China), and underwent surgery between September 2021 and June 2023. Our center is the Diagnosis and Treatment Center for Colorectal Diseases in Hebei Province. In 2023, the number of colorectal cancer surgeries was 1926, of which about 90% were laparoscopic surgeries, with an average of 54 surgeries per surgeon. Inclusion criteria: (1) all surgical procedures were conducted by an expert surgeon (Y.Y. Ding) who performed laparoscopic radical surgery for left-sided colorectal cancer; (2) patients with preoperative imaging data, such as whole abdominal enhanced CT with feasible 3D vascular reconstruction, were available; (3) patients with complete high-definition surgical videos, and clear images of submesenteric vessels; (4) patients with surgical videos including a 1 cm sterile equal-length scale prepared in advance using Johnson silk thread as a means of measurement, the surgical video reviewers were two experienced surgeons: W.B. Niu and X.H. Hu. Exclusion criteria: (1) patients who suffered intraoperative bleeding and contamination of the operative field that made it impossible to determine the type and composition of blood vessels; (2) patients who failed to undergo radical resection due to late tumour staging; (3) patients who required laparoscopic conversion to laparotomy; (4) patients who underwent emergency surgery, without preoperative data such as whole abdominal enhanced CT.

In this study, we enrolled 200 patients and collected information on age, sex, body mass index (BMI), American Society of Anaesthesiology (ASA) classification score, primary tumour location, histopathological stage (AJCC TNM stage), and operation time. This research followed the ethical guidelines of the World Medical Association (WMA; Declaration of Helsinki). Approval for the present research was obtained from the Ethics Committee of the Fourth Hospital of Hebei Medical University (approval number 2022KY420, retrospectively registered), and all patients provided informed, written consent for the clinical study.

### The pattern of the IMA and its branches

**Figure 1:**
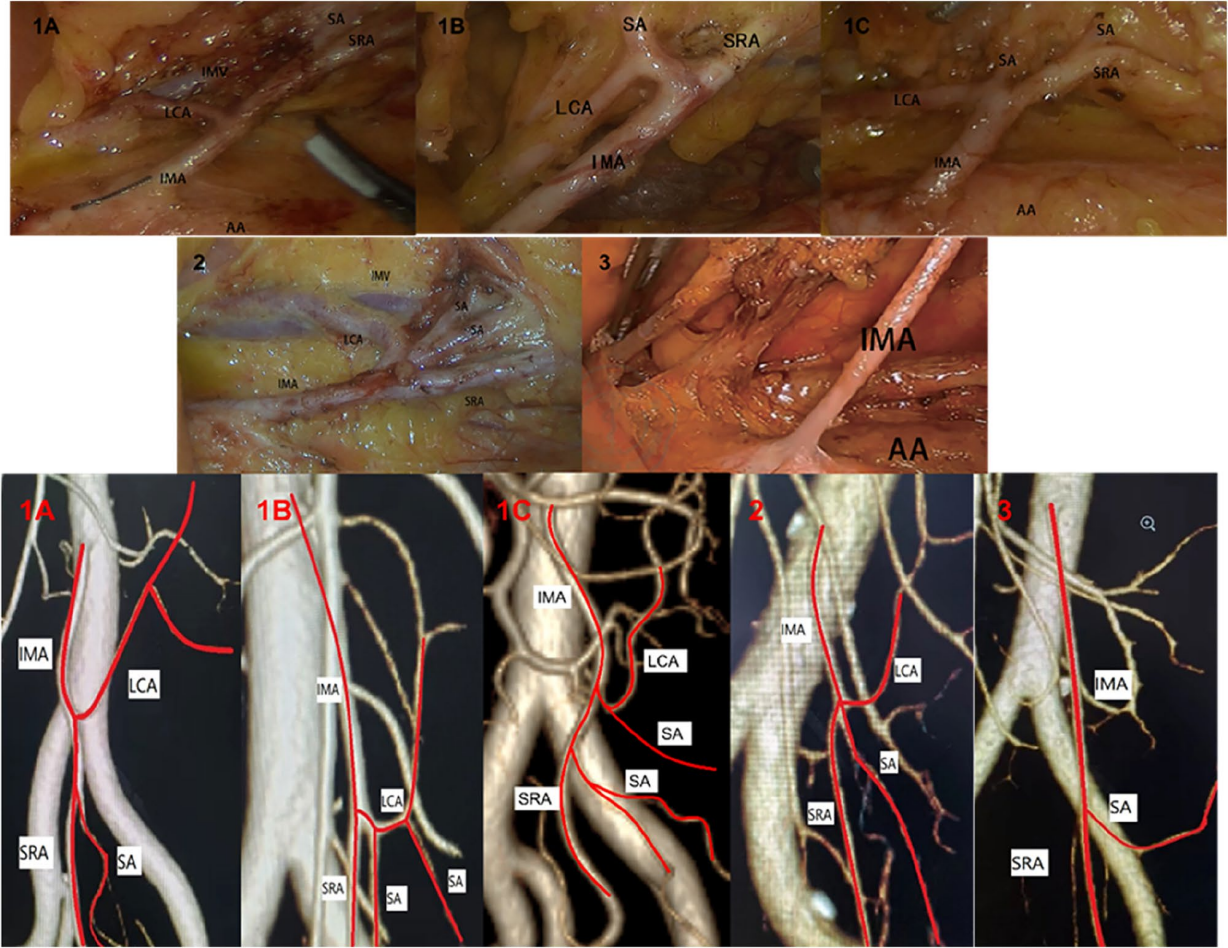
The branching pattern of the IMA is divided into five types. *IMA* inferior mesenteric artery, *LCA* left colic artery, *SA* sigmoid colon, *SRA* superior rectal artery, *IMV* inferior mesenteric vein, *AA* abdominal aorta.

In this study, the pattern of the IMA and its branches were observed and classified according to the classification described by McSweeney et al.^14^. We classified the branching patterns of the IMA into five types (1A, 1B, 1C, 2, and 3). First, we distinguished whether the IMA was divided into two or three branches. When the IMA divides into two branches (type 1), it must be assessed for whether the sigmoid arteries (SAs) arise from the common trunk with the superior rectal artery (SRA) (type 1A), the common trunk with the LCA (type 1B), or from both (type 1C). In type 2, the IMA divides into three branches at a common origin. Type 3 is classified as the absence of the LCA (Fig. 1).

### Observation indicators and measurement methods

By reviewing the surgical videos of all patients enrolled, all the enrolled patients had 253 lymph nodes dissected during the surgical procedure for IMA. The scope of dissection was as follows: the inner boundary was the trunk area between the IMA emitting point and the LCA emitting point, the lateral border was the medial border of the IMV, the caudal border was the point from the LCA to its intersection with the IMV, and the cephalic border was at the level of the IMA root^15^. (1) Intraoperatively, the course of the IMA trunk and its branches were observed and classified; (2) Intraoperative measurement of the distance between the origin of the IMA and the point of emanation of the LCA (D cm) using a filament; (3) The relationship between the position of the LCA and the IMV and the distance from the origin of the LCA to the intersection of the LCA and the IMV were observed and measured.

3D-CT vascular imaging was performed using a Philips ICT 256-layer spiral CT scanner. After fasting for at least 4 h before the examination, the patients were placed in a supine position, underwent scanning from the top of the diaphragm to the level of the sciatic tuberosity, and received an injection of 80–90 mL of iopromide via high-pressure syringe into the median elbow at a flow rate of 4.5 mL/s with an arterial phase delay of 24–28 s. The multilayer spiral CT scanner generated 0.75 mm slices and reconstructed 0.5 mm images, and the images were analysed using the Daijia A-Site V4.0 system (DJ HealthUnion Systems Corp., Shanghai, China) for image processing and analysis using 3D volume rendering technology. We used the 3D-CT vascular reconstruction technique to analyse the preoperative images of all the enrolled patients and selected the images of the arterial enhancement phase for the following observation and measurement. (1) to observe and record the pattern of the IMA and its branches; (2) to display the longest angle on 3D images to measure the distance between the root of the IMA and the beginning of the LCA (d cm); (3) to observe the relationship between the position of the LCA and IMV on the axial 2D images; (4) to measure the distance from the origin of the IMA to the bifurcation of the AA; and to observe the vertebral level of the origin of the IMA.

### Statistical methods

IBM SPSS Statistics software (version 26.0; IBM Corporation, Armonk, NY, USA) was used for data analysis. The quantitative data satisfied the t-test of normal distribution, and the results were expressed as the mean ± standard deviation, while those that did not satisfy a normal distribution were expressed by the Mann‒Whitney U test, and the results were expressed as the median (min–max). The χ^2^ test or Fisher’s exact probability test was used for count data. The test level was α = 0.05, and P < 0.05 was considered a statistically significant difference.

### Ethical approval

Approval for the present research was obtained from the Ethics Committee of the Fourth Affiliated Hospital of Hebei Medical University (approval number 2022KY420, retrospectively registered), and all patients provided informed, written consent for the clinical study.

## Results

### The patterns of IMA and its branches

The percentages of the five types of IMA branching patterns (types 1A, 1B, 1C, 2, and 3) are shown in Table 1. From the surgical videos, we found that type 1A (47.0%) was the most frequent, followed by type 1B (30.5%), and types 2 and 1C accounted for 13.5% and 3.5%, respectively. Type 3, which lacks a LCA, was found in only 11 patients (5.5%). In contrast, from the analysis of the preoperative images, we conclude that the proportions of types 1A, 1B, 1C, 2, and 3 are 45.5%, 29.5%, 4.0%, 15.5%, and 5.5%, respectively. The results of the two methods are similar, with no statistically significant differences (p = 0.979).

**Table 1:** Relationship of IMA branch patterns between the surgical video group and the 3D revascularization group.

Group	The patterns of the IMA branch					*p* value
	Type 1A (%)	Type 1B (%)	Type 1C (%)	Type 2 (%)	Type 3 (%)	
Surgical video group	94 (47.0)	61 (30.5)	7 (3.5)	27 (13.5)	11 (5.5)	0.979
3D revascularization group	91 (45.5)	59 (29.5)	8 (4.0)	31 (15.5)	11 (5.5)	

We also performed a univariate analysis of the clinical characteristics of patients between branch types in the surgical video group, including age, sex, BMI, ASA score, location of the primary tumour, histopathological stage (AJCC TNM stage) and operation time. There was no statistically significant difference in the clinical characteristics between the patterns of the IMA in the surgical video group (Table 2).

**Table 2:** Relationship between patient clinical characteristics and IMA vascular patterns in the surgical video group.

	Type 1A (n = 94)	Type 1B (n = 61)	Type 1C (n = 7)	Type 2 (n = 27)	Type 3 (n = 11)	*p* value
Age, years	51 (45–86)	67 (54–69)	59 (55–62)	58 (49–71)	66 (62–74)	0.729
Sex ratio (male/female)	53/41	38/23	2/5	17/10	3/8	0.166
BMI, kg/m^2^	22.03 ± 3.86	20.18 ± 3.02	24.26 ± 2.09	28.78 ± 1.99	22.92 ± 1.29	0.872
ASA score	2.07 ± 0.26	2.21 ± 0.47	2.75 ± 0.50	2.26 ± 0.45	2.50 ± 0.58	0.102
AJCC TNM stage						0.536
0	2	4	0	2	1	
I	11	12	2	2	3	
IIA, IIB, IIC	47	29	3	5	4	
IIIA, IIIB, IIIC	33	13	1	14	3	
IV	1	3	1	4	0	
Operative time, min	210 ± 66	211 ± 65	218 ± 64	198 ± 40	176 ± 50	0.784
Location of the main tumour						0.219
Descending colon	4	4	1	3	0	
Sigmoid colon	20	21	3	5	4	
Rectum	70	36	3	19	7	

### Analysis of the distance from the root of the IMA to the origin of the LCA

We measured the distance from the root of the IMA to the origin of the LCA in all patients enrolled (except for the type without LCA), using two methods (D for intraoperative filament measurements; d for preoperative imaging), with D cm and d cm ranging from 1.84–6.62 cm (median 3.66 cm) and 1.85–6.52 cm (median 3.67 cm), respectively. Eleven patients with no LCA were excluded from this analysis. In types 1A, 1B, 1C and 2, the range of D is 1.90–6.62 cm (median 3.60 cm), 2.16–6.25 cm (median 3.76 cm), 1.84–4.85 cm (median 3.60 cm) and 1.90–6.25 cm (median 3.53 cm). d was 1.85–6.52 cm (median 3.10 cm), 2.11–6.34 cm (median 3.83 cm), 1.92–5.10 cm (median 3.57 cm) and 1.94–6.22 cm (median 2.98 cm). We performed a statistical analysis of the conclusions drawn from the two methods, and there was a significant difference between the two methods (p < 0.001) (Table 3).

**Table 3:** Relationship of IMA length between the surgical video group and the 3D revascularization group.

Group	The length of IMA (mean ± SD) (cm)	*Z*	*p* value
Surgical video group	3.71 ± 0.97	3.629	< 0.001
3D revascularization group	3.76 ± 0.99		

### The adjacency relationship between LCA and IMV

We examined the adjacency relationship between the LCA and the IMV, and eleven patients with no LCA were not included in this analysis. Intraoperative video observation concluded that 65.6% (n = 124) of LCAs were aligned anteriorly from the IMV and that 34.4% (n = 65) were aligned posteriorly. The LCA travelled medially to the IMV in 57.7% (n = 109) of patients, and the LCA travelled laterally to the IMV in 42.3% (n = 80) of patients. In the analysis of the preoperative images, the proportion of different positional relationships was consistent with the former.

We also measured the distance from the origin of the LCA to the intersection of the LCA and IMV using intraoperative silk thread, with the length of 0.64–4.29 cm (median 3.03 cm), 0.87–4.35 cm (median 3.20 cm), 1.32–4.28 cm (median 3.11 cm) and 1.65–3.69 cm (median 3.00 cm) in types 1A, 1B, 1C and 2, respectively, with no statistically significant difference among the groups (p = 0.994). The distance between the LCA and IMV was not measured on CT images in this study, as the relationship between the LCA and IMV could not be well displayed in 3D-CT vascular reconstruction.

### Starting point of IMA

This study utilized preoperative CT images of the IMA origin because it was difficult to determine the vertebral level of origin during the operation. Of the observed IMAs, 73.5% (n = 147) came from the L3 level, 20.0% (n = 40) originated from the L4/5 level, and 6.5% (n = 13) originated from the L3 level or above (with the highest point of emanation located at the L1/2 level). The D_IMA_, which refers to the distance from the point of origin to the AA bifurcation, ranged from 2.63 to 6.74 cm (with a median of 4.33 cm).

### The relationship between IMA length and clinical characteristics of patients

In the surgical video group, the length of the IMA ranged from 1.84 to 6.62 cm, which we divided into two groups according to the median length of 3.66 cm. Univariate regression analyses of the length of the IMA and the patients' clinical characteristics showed only body weight was associated with the length of IMA (Table 4).

**Table 4:** Relationship between IMA length and clinical characteristics in the surgical video group. * Indicates significant differences.

	IMA ≤ 3.66 cm	IMA > 3.66 cm	Univariate analysis
	n = 95	n = 94	*p *value
Age, years	63 (30–86)	64 (43–78)	0.384
Sex ratio (male/female)	1.32 (54/41)	1.47 (56/38)	0.872
Height, cm	163.5 ± 7.7	168.2 ± 6.8	0.284
Body weight, kg	68.5 ± 10.6	75.6 ± 9.5	0.013*
BMI, kg/m^2^	23.5 ± 3.8	25.1 ± 3.5	0.081
AJCC TNM stage			0.856
0	3	5	
1	12	15	
2	45	39	
3	31	30	
4	4	5	
Location of the main tumour			0.090
Descending colon	9	3	
Sigmoid colon	20	29	
Rectum	66	62	

Meanwhile, we statistically analysed the relationship between IMA length and patients' clinical characteristics in the 3D-CT revascularization group, which was divided into two groups according to their median length of 3.67 cm, and the results showed that a longer D_IMA_ and greater body weight were independently associated with a longer length of the IMA (Table 5).

**Table 5:** Relationship between IMA length and clinical characteristics in the 3D revascularization group. * Indicates significant differences.

	IMA ≤ 3.67 cm	IMA > 3.67 cm	Univariate analysis	Multivariate analysis		
	n = 94	n = 95	*p* value	CI95%	OR	*p* value
Age, years	63 (30–86)	64 (43–78)	0.384			
Sex ratio (male/female)	1.24 (52/42)	1.5 (57/38)	0.783			
Height, cm	163.8 ± 7.4	168.1 ± 7.2	0.302			
Body weight, kg	68.9 ± 9.9	75.5 ± 9.6	0.015*	0.276–0.898	0.499	0.008*
BMI, kg/m^2^	23.4 ± 3.4	25.2 ± 3.7	0.076			
D_IMA_ ,mean ± SD, ≤ 4.33 cm vs. > 4.33 cm	4.22 ± 0.65	4.59 ± 0.82	0.005*	0.204–0.759	0.393	0.004*
AJCC TNM stage			0.959			
0	3	5				
1	13	14				
2	43	41				
3	31	30				
4	4	5				
Location of the main tumour			0.166			
Descending colon	9	3				
Sigmoid colon	22	27				
Rectum	63	65				

## Discussion

In this retrospective observational study, we used two methods to classify IMA branch patterns more accurately: reviewing surgical videos and preoperative imaging data. There were no statistically significant differences in the proportions of each type derived from the two methods. Of all types, type 1A (47.0%) was the most common. At present, most of the research results on IMA branch patterns are derived from multiple spiral CT vascular reconstruction techniques, with reported proportions of types 1A, 1B, 2, and 3 ranging from 36 to 72.8%, 9–46%, 8–44.7%, and 0.9–6%^9,10,16–18^, respectively. The results of these studies are similar to those of our study. Zhang et al. ^19^ used the method of directly observing the pattern of the inferior mesenteric vessels intraoperatively and measuring the relative distances between the vessels. In his report, the probability of type 1A was 59.5%, while that of type 1B was 29.2%, type 2 was 8.5%, and type 3 was 2.8% (lacking LCA), which was generally consistent with the results of our study. In the present research, only Sinkeet et al. ^20^ and Griffiths et al. ^16^ reported the probability of the occurrence of type 1C, which were 1.75% and 15%, respectively. The results of our study are similar to those of Sinkeet et al. ^20^. In the present study, few studies have studied the classification of type 1C, and most researchers classify it as type 1A or 1B. In our study, we found that the SA was arising from the common trunk both the LCA and the SRA, so we classified it as type 1C. Through 3D-CT vascular reconstruction, we found that this blood vessel originated from the LCA and finally took shape in the sigmoid colon, not the descending branch of the LCA. Therefore, we believe that type 1C should not be ignored in the vascular classification of the IMA. Although there was no statistically significant difference between the proportions of each type derived from the two methods in our study, three patients were classified as type 2 in the 3D-CT vascular reconstruction, but during the operation, we found that the actual classification was type 1A. Considering that 3D-CT vascular reconstruction is based on computer software processing when the distance between the emanation point of the SA and LCA is short, there may be errors between the 3D vascular model and the actual vascular classification.

Among the reports on the length of the IMA, Singh et al. ^21^ and Sinkeet et al. ^20^ used autopsy, and the average length between the IMA and LCA was 2.5–4.5 cm. Most studies use the method of 3D-CT vascular reconstruction for measurement, and the length range is 1.01–8.22 cm^9,10,17,18,22–24^. Zhang et al. ^19^ used intraoperative silk threads to measure vessel length, and the average length measured was 1.50–6.53 cm. 3D-CT vascular reconstruction is performed by computer software, but its accuracy has not been confirmed compared to direct intraoperative measurements. In our study, for the first time, the length of the IMA was compared using both retrospective laparoscopic surgical video and 3D-CT vascular reconstruction, and there was a significant difference (p < 0.001), indicating that there was a certain difference between the data obtained by 3D-CT vascular reconstruction and the actual situation of patients. However, in this study, the maximum difference between the two methods was 0.93 cm, among the 200 patients, 182 (96.3%) had a length difference of 0–0.5 cm, which we think is an acceptable error for intraoperative vascular anatomy measurements. We cannot deny the value of 3D-CT vascular reconstruction technology, it can provide a reference value for surgeons to predict the location of blood vessels in the colon in advance and clarify the anatomical distance of blood vessels.

Based on multivariate regression in this study, IMA length was found to be independently associated with body weight. Therefore, surgeons can expect a shorter IMA and less stress when performing low-level ligation in patients with lighter body. Bleeding from blood vessels during left-sided colorectal cancer surgery mostly occurs as a result of treatment of veins. Therefore, we first summarized the positional relationship between the LCA and IMV, and the results were similar to those of Murono et al. ^17^, in that we found no difference between intraoperative observation data and imaging data. Second, there are few studies on the distance between the IMV and LCA. During the operation, we measured the distance from the starting point of the LCA to the intersection of the LCA and IMV. The results were similar to those of Zhang et al. ^19^, who found that the distance between them was not affected by IMA typing.

The importance of the IMA branch pattern is largely related to the increased number of colorectal cancers treated with laparoscopic techniques^10,19^. For D3 lymph node dissection in laparoscopic radical surgery for sigmoid and rectal cancer, the purpose is not only to remove the tumour, but also to remove the lymph nodes around the blood vessels and the nerve and vascular sheath associated with the blood vessels^25^. This procedure is more difficult and time-consuming^11,24^. The level of arterial ligation in rectal cancer surgery has always been controversial. Typically, we refer to ligation below the point of LCA emanation as low ligation. Many surgeons prefer high ligation of the IMA to facilitate complete lymphadenectomy^11^; however, low ligation may be beneficial because it provides a better blood supply to the anastomosis, thereby reducing the probability of anastomotic fistula development^3^. The occurrence of anastomotic fistula is caused by a variety of factors, such as the blood supply of the distal and proximal intestinal canal of the anastomosis, the tension of the anastomosis, the excessive pressure in the proximal intestinal canal of the anastomosis, the intestinal preparation before an operation and the patency of the anastomosis ^26^. Although the blood supply level cannot completely determine the occurrence of anastomotic fistula, all the above factors can be considered to find a solution; only the blood supply level is determined by whether the LCA is retained during the operation, and it is irreversible. A rich blood supply of the anastomosis can provide a good anastomotic growth environment. Therefore, we hope to summarize the relationship between the location and length of the IMA and its branches through our study, and to demonstrate the accuracy of 3D-CT vascular reconstruction technology, to provide a reference for intraoperative blood vessel ligation for surgeons.

The main findings of this study are to verify the applicability and precision of preoperative 3D-CT vascular reconstruction of IMA and its branches by comparing with intraoperative surgery videos. If surgeons are aware of the length and branching pattern of IMA, and the surrounding position of IMA and LCA preoperatively, they can precisely estimate the difficulties of the surgery, reduce the risk of intraoperative bleeding and make individualized operational strategies. In this condition, operation time would be shortened and fast track surgery could be more easily achieved. This study also provides a basis for future development of human based 3D-CT vascular reconstruction.

This study has several limitations. First, this study is a single-centre, retrospective study, and there are certain regional limitations. Second, the number of cases included in this study is relatively small, and more cases need to be included to further improve the reliability. Third, although in this study, the preoperative vascular length measurement was independently measured by two researchers and the two values were averaged to reduce errors, the measurement errors still exist and are inevitable. Whether 3D-CT vascular reconstruction technology can reduce bleeding or be more conducive to lymph node dissection, needs further study. Meanwhile, multi-centre and long-term follow-up prospective studies are needed to further elucidate the accuracy of 3D-CT vascular reconstruction and its value in surgery, and these are also our next research direction.

## Conclusion

There was only a significant difference in the length of the IMA between the 3D-CT vascular reconstruction and intraoperative observation data. 3D-CT vascular reconstruction can help surgeons predict the distance and position relationship of the IMA and its branches preoperatively, thereby shortening the operation time, reducing the occurrence of complications such as bleeding, and guaranteeing the safety of low-level ligation.

## Data Availability

The datasets used and analysed during the current study are available from the corresponding author upon reasonable request.
